# A Clinical Evaluation of Cardiovascular Emergencies: A Comparison of Responses from ChatGPT, Emergency Physicians, and Cardiologists

**DOI:** 10.3390/diagnostics14232731

**Published:** 2024-12-04

**Authors:** Muhammet Geneş, Bülent Deveci

**Affiliations:** Cardiology Residency, Department of Cardiology, Sincan Training and Research Hospital, Ankara 06930, Turkey; bulentdeveci@hotmail.com

**Keywords:** acute cardiological cases, artificial intelligence (AI), ChatGPT, clinical decision support

## Abstract

**Background:** Artificial intelligence (AI) tools, like ChatGPT, are gaining attention for their potential in supporting clinical decisions. This study evaluates the performance of ChatGPT-4o in acute cardiological cases compared to cardiologists and emergency physicians. **Methods:** Twenty acute cardiological scenarios were used to compare the responses of ChatGPT-4o, cardiologists, and emergency physicians in terms of accuracy, completeness, and response time. Statistical analyses included the Kruskal–Wallis H test and post hoc comparisons using the Mann–Whitney U test with Bonferroni correction. **Results:** ChatGPT-4o and cardiologists both achieved 100% correct response rates, while emergency physicians showed lower accuracy. ChatGPT-4o provided the fastest responses and obtained the highest accuracy and completeness scores. Statistically significant differences were found between ChatGPT-4o and emergency physicians (*p* < 0.001), and between cardiologists and emergency physicians (*p* < 0.001). A Cohen’s kappa value of 0.92 indicated a high level of inter-rater agreement. **Conclusions:** ChatGPT-4o outperformed human clinicians in accuracy, completeness, and response time, highlighting its potential as a clinical decision support tool. However, human oversight remains essential to ensure safe AI integration in healthcare settings.

## 1. Introduction

Cardiovascular diseases (CVDs) remain one of the leading causes of death worldwide. In 2019, an estimated 17.9 million people died from CVDs, accounting for 32% of all global deaths. Of these deaths, 85% resulted from acute cardiovascular events. More than three-quarters of CVD-related deaths occur in low- and middle-income countries, and 38% of the 17 million premature deaths attributed to non-communicable diseases are linked to CVDs [1]. These statistics emphasize the importance of diagnosing cardiovascular emergencies as early as possible and initiating effective treatment promptly. In acute cardiac emergencies, rapid diagnosis and intervention are essential to reducing morbidity and mortality. Early intervention enables more effective patient management and provides a crucial opportunity to lower mortality rates, particularly in high-risk patients. Thus, the principle of ‘Time is Life’ serves as a cornerstone in the management of cardiovascular emergencies [2]. The rapid evolution of knowledge, technology, and healthcare systems plays a vital role in saving lives during cardiovascular emergencies. In this context, artificial intelligence (AI) has emerged as a transformative tool, with the potential to enhance decision-making processes, optimize workflows, and improve patient outcomes in the management of cardiological emergencies [3].

AI is a field of computer science dedicated to creating systems capable of mimicking human cognitive functions [4]. These systems independently process information and perform tasks by gathering data from their digital environments. Machine learning, a key component of artificial intelligence, extracts patterns and insights from large datasets and uses this information to make predictions and develop decision support systems. Natural Language Processing (NLP), a subset of artificial intelligence, aims to analyze and simulate human language by processing, understanding, and generating text and speech data. In recent years, the scope of AI has expanded to encompass sectors such as diagnostic healthcare services, the Internet of Things, and the development of smart devices [5,6]. ChatGPT, created by OpenAI, is designed to simulate natural language responses based on textual input using deep neural network methodologies. As part of the Generative Pre-trained Transformer (GPT) model lineage, ChatGPT is regarded as one of the most comprehensive language models available to the public. By analyzing an extensive range of textual information, it proficiently discerns linguistic elements, enabling it to generate relevant and nuanced dialogues in diverse contexts [6]. While AI has been present for some time in customer services and knowledge management, its use in healthcare and medical research is rapidly expanding. ChatGPT, as an AI language model based on deep learning, holds significant potential in clinical decision support. With its ability to generate human-like text responses, it can analyze patient data, provide evidence-based recommendations, and assist clinicians in treatment processes. Its capabilities in language comprehension and generation make it applicable to various domains, including diagnosis, treatment planning, and patient management [7]. However, the responsible integration of this technology into healthcare systems requires consideration of limitations such as biases and the necessity of human oversight.

The primary objective of this study is to assess the accuracy, reliability, and speed of AI in clinical decision-making processes in critical scenarios, such as cardiological emergencies. Specifically, it aims to explore how ChatGPT can support clinical decision-making by working in harmony with emergency medicine and cardiology specialists, thereby enhancing patient care outcomes. Additionally, the study seeks to examine both the advantages and limitations of AI in decision-making processes, aiming to establish the necessary conditions for its safe and effective integration into clinical practice. This work also aspires to provide valuable insights that will guide future academic investigations.

## 2. Materials and Methods

### 2.1. Study Design

The study was designed to evaluate the performance of the ChatGPT-4o model, a natural language processing and comprehension tool developed by OpenAI (San Francisco, CA, USA), in managing cardiological emergencies, following the standards outlined in current cardiology guidelines. It aimed to assess the feasibility of ChatGPT-4o as a clinical decision support tool by examining its accuracy, reliability, and potential role as an educational resource for healthcare professionals. The study utilized 20 methodical clinical scenarios derived from up-to-date cardiology guidelines. This study did not need ethical approval as no patient-level data were used.

### 2.2. Study Setting

In this study, 20 open-ended clinical scenarios focusing on cardiological emergencies were presented to ChatGPT-4o, three emergency physicians, and three cardiologists. The primary aim of the study was to compare the clinical decision-making performance of participants based on these scenarios. The cardiologists and emergency physicians who participated in the study were all specialist physicians with at least five years of clinical experience in their respective fields. No residents or trainee doctors were included among the participants. This level of expertise aimed to ensure that evaluations were conducted accurately and reliably while providing a senior perspective on clinical decision-making processes. The 20 clinical scenarios used in this study were designed to encompass a broad spectrum of acute cardiological emergencies. The cases included a range of conditions such as acute coronary syndromes and myocardial infarction (6 cases), pulmonary embolism (2 cases), hypertensive crises (1 case), cardiac tamponade and trauma (2 cases), tachycardia and arrhythmias (4 cases), heart failure (2 cases), and drug-related conditions and coagulopathy management (2 cases) (Supplementary Materials). These scenarios were compiled from board examination questions, internationally recognized guidelines (e.g., ESC and AHA), published literature, and real-world clinical cases. Each case was selected to ensure clinical diversity and included varying levels of complexity, ranging from fundamental diagnostic and treatment processes to intricate management scenarios. Twelve cases were categorized as basic, focusing on rapid diagnosis and emergency treatment, while four were of moderate complexity, and the remaining four involved advanced clinical decision-making processes with complex variables. The complexity of each case was determined based on the diagnostic and therapeutic challenges it posed, as assessed by an expert panel. Simple cases included scenarios such as STEMI management, which could be diagnosed and treated easily using standard protocols. Moderately complex cases required evaluating multiple diagnostic criteria or discussing alternative treatment options, such as selecting the appropriate imaging modality for pulmonary embolism. Complex cases involved integrating multiple clinical factors and required advanced decision-making skills. All scenarios were reviewed and revised by two expert panelists to enhance their real-world applicability, and were subsequently used to fairly compare the performance of physicians at varying levels of expertise with that of the AI system.

Three emergency physicians and three cardiologists voluntarily participated in the study. Each participant was assessed independently and was unaware of the presence of other participants during the evaluation to prevent potential bias. The clinical scenarios were presented to the emergency medicine and cardiology specialists in an oral examination format. This method was chosen to objectively measure response times and standardize time management across participants. All participants were expected to solve the scenarios quickly and accurately, reflecting real-world decision-making processes. No time limits were imposed, and no feedback or comments were provided at the end of the assessment. The response times of participants and the ChatGPT model were measured using a standardized stopwatch method. A timer was initiated immediately after each question was presented and stopped upon the completion of the response. Following the completion of all questions, the cumulative response times for each participant and the AI model were calculated by summing the individual response times across all questions. This cumulative time was subsequently analyzed to compare the efficiency of human participants and the ChatGPT model. Informed consent was obtained from all participants. The same clinical scenarios were presented to the ChatGPT-4o model in written format twice, with the system being restarted between the attempts, and its responses were recorded for subsequent analysis. ChatGPT-4o received no prior guidance or exposure to the questions before the evaluation, ensuring a fair comparison with human participants. This design aimed to establish equal conditions for both human participants and the AI model, focusing on clinical accuracy, completeness, and response efficiency.

### 2.3. ChatGPT-v4o and Human Analysis

In this study, ChatGPT-4o, a natural language processing and comprehension tool developed by OpenAI, was employed to assist with complex decision-making processes between 1 August and 30 August 2024. The primary objective was to evaluate the accuracy and comprehensiveness of the AI’s responses to cardiological emergency scenarios. The Likert scale, a widely recognized research tool, was utilized to assess response accuracy based on a six-point scale: 1 = Completely false, 2 = Mostly false, 3 = Both true and false, 4 = More true than false, 5 = Almost completely true, 6 = True. Completeness was evaluated using a three-level scoring system: “Inadequate” for responses addressing only partial aspects of the problem with missing or incomplete critical components, “Adequate” for responses covering all facets of the question with the minimum required information, and “Comprehensive” for responses exceeding expectations by providing additional context or insights. The study included 20 clinical scenarios ranging from straightforward diagnostic cases to complex presentations, designed to reflect real-world acute cardiovascular emergencies. Participants were tasked with identifying appropriate diagnostic and management steps in line with current cardiology guidelines.

ChatGPT-4o’s responses were independently reviewed by two experienced cardiologists (M.G. and B.D.) based on their adherence to guidelines, accuracy, and relevance to the latest cardiology standards. Discrepancies between reviewers were resolved through consensus discussions. The study sought to objectively compare ChatGPT-4o’s performance with three key metrics: accuracy, defined as the percentage of correct responses; consistency, referring to the ability to provide uniform answers to similar scenarios; and comprehensiveness, which assessed whether responses incorporated the necessary depth and breadth of information in alignment with cardiology guidelines.

A “success” metric was developed to determine whether a response was clinically accurate, comprehensive, and guideline-adherent. For a response to be deemed “successful”, it required a Likert scale score of at least 4 for accuracy and to be categorized as either “Adequate” or “Comprehensive” in completeness. Success was further measured not only by the correctness of the response but also by its ability to address the clinical context, offering appropriate management and treatment plans. This methodology ensured a structured and unbiased evaluation of the AI model’s performance in managing cardiological emergencies.

#### Data Analysis

In this study, the degree accuracy of responses provided by ChatGPT-4o cardiologists and emergency physicians for 20 cardiological emergency scenarios, as well as their response times, were evaluated. Descriptive and inferential statistical methods were employed to determine differences in performance between the three groups. The correct and incorrect responses to each scenario were analyzed to compare the performance across groups. The mean response times of emergency medicine and cardiology specialists were considered in comparison with ChatGPT 4o Before proceeding with the analysis of differences between groups, the assumption of normal distribution was evaluated using the Shapiro–Wilk test. The differences between groups were assessed using the Kruskal–Wallis H test and post hoc analysis with the Mann–Whitney U test at a 95% confidence interval. Inter-rater success rate assessment was made using Chi-square Fisher’s Exact Test. In all analyses, a *p*-value of < 0.05 was considered the threshold for statistical significance. Data analysis was conducted using SPSS software, version 26, provided by IBM Corporation, Armonk, NY, USA.

## 3. Results

### Descriptive Findings

Descriptive findings related to the raters’ “Success”, “Accuracy”, and “Completeness” values are presented in Table 1.

In Table 1, a cross-tabulation analysis was performed to evaluate the proportions of correct responses provided by emergency physician, cardiologists, and the ChatGPT-4o model. The correct response rates among emergency physician ranged between 65.0% (*n* = 13) and 80.0% (*n* = 16). Cardiologists and ChatGPT-4o demonstrated the best performance, each with a 100% (*n* = 20) correct response rate. The analysis revealed that the success rates of cardiologists and ChatGPT-4o were statistically significantly higher than those of emergency physicians (χ^2^ = 22.909, *p* < 0.001).

The accuracy scores for the emergency physicians ranged between 4.06 (±1.30) and 4.53 (±0.94). Emergency physician C achieved the highest accuracy score, while physician B had the lowest average. The mean accuracy score for the group of emergency physician was calculated as 4.14 (±1.15). The cardiologists’ accuracy scores were higher; cardiologist B had the highest average score, with the group’s mean score found to be 5.75 (±0.30). ChatGPT-4o achieved the highest accuracy score with 5.85 (±0.37) (Table 1).

The completeness scores for emergency physician ranged between 1.41 (±0.51) and 1.65 (±0.61). Emergency physician A had the lowest average completeness score, while physician C achieved the highest average. The mean completeness score for the group of emergency physicians was calculated as 1.64 (±0.71). Cardiologists’ completeness scores ranged between 2.20 (±0.52) and 2.30 (±0.47); cardiologists A and B both had an average score of 2.20 (±0.41), while cardiologist C reached the highest completeness score with 2.30 (±0.47). ChatGPT-4o achieved a completeness score of 2.75 (±0.55) (Table 1).

Table 2 provides a summary of the cumulative response times of emergency physicians, cardiologists, and the AI system ChatGPT-4o, expressed in minutes. The response times for emergency physicians ranged from 32.53 min (Physician C) to 33.64 min (Physician A), with an average of 33.1 min. The variation in response times among this group was minimal, indicating consistency in their performance. Cardiologists demonstrated slightly shorter response times compared to emergency physicians, ranging from 27.55 min (Cardiologist C) to 29.12 min (Cardiologist A), with an average of 28.32 min. This suggests greater efficiency in completing clinical scenarios. The AI system achieved the fastest response time, averaging 11.27 min. This was substantially quicker than both the emergency physicians and cardiologists, reflecting the AI’s computational speed and efficiency in processing clinical data.

For the accuracy scores, the ChatGPT-4o group had a mean score of 5.85 (±0.37), with a rank average of 40.80. The emergency physicians group had a mean accuracy score of 4.14 (±1.15), with a rank average of 11.83. For cardiologists, the mean accuracy score was 5.75 (±0.30), with a rank average of 34.10. The Kruskal–Wallis H test revealed a significant difference among the groups (H = 33.802, *p* < 0.001). Post hoc analysis showed that the difference between ChatGPT-4o and emergency physicians was statistically significant, with ChatGPT-4o achieving higher accuracy scores than emergency physicians (1 < 2, *p* < 0.001). Additionally, a significant difference was found between emergency physicians and cardiologists, with cardiologists scoring higher than emergency physicians (2 < 3, *p* < 0.001) (Table 3).

In terms of completeness scores, the ChatGPT-4o group had a mean score of 2.75 (±0.55), with a rank average of 42.95. The emergency physicians group had a mean completeness score of 1.64 (±0.71) and a rank average of 14.86. For the cardiologists, the mean score was 2.23 (±0.36), with a rank average of 29.23. The Kruskal–Wallis H test revealed significant differences among the groups (H = 27.671, *p* < 0.001). Post hoc analysis showed a statistically significant difference between ChatGPT-4o and emergency physicians, with ChatGPT-4o achieving higher completeness scores (1 < 2, *p* < 0.01). Additionally, a significant difference was observed between emergency physicians and cardiologists, with cardiologists scoring higher than emergency physicians (2 < 3, *p* < 0.01). Furthermore, there was a statistically significant difference between ChatGPT-4o and cardiologists, with ChatGPT-4o outperforming cardiologists in terms of completeness scores (1 > 3, *p* < 0.05) (Table 3).

Table 4 compares the accuracy and completeness scores among ChatGPT-4o, cardiologists, and emergency physicians. ChatGPT-4o achieved significantly higher scores than both emergency physicians (*p* < 0.001) and cardiologists (*p* < 0.001) in accuracy and completeness. Cardiologists outperformed emergency physicians in completeness (*p* = 0.021), while no significant difference was observed between them in accuracy (*p* = 0.184).

**Figure 1:**
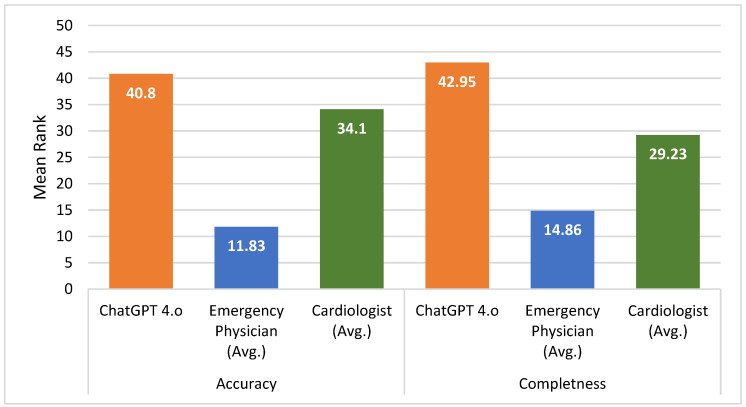
Findings regarding the comparison of accuracy and completeness among raters.

These findings show that ChatGPT-4o achieved significantly higher success, accuracy, and completeness scores compared to emergency physicians and cardiologists, highlighting differences in performance among the groups. The high inter-rater agreement, with a Cohen’s kappa score of 0.92, further supports the reliability of these assessments (Figure 1).

## 4. Discussion

The use of AI technologies in clinical decision support systems represents a significant and innovative development in healthcare [8]. In emergency department settings, physicians often work under stressful conditions, with long shifts and increasing patient loads, requiring them to make rapid decisions. However, it is well known that faster decision-making can sometimes compromise accuracy. These demanding working conditions—combined with distractions, difficulties in accessing information promptly, and time pressure—can increase the likelihood of diagnostic errors. Studies show that the rate of diagnostic errors in emergency departments can vary between centers, reaching up to 64% [9]. Delays in treatment, rising healthcare costs, elevated patient anxiety, and a decline in trust toward healthcare services are other adverse outcomes that may result from these challenges. Furthermore, timely access to specialized consultations is not always available in emergency situations, which can negatively impact patient care.

Over the past few years, there has been an increasing number of studies examining the potential of AI models in triage and diagnostic accuracy within emergency departments, comparing their performance to that of human clinicians [10]. Many of these studies have highlighted significant benefits of AI-based applications, such as faster clinical decision-making processes and reduced task completion times, aligning with the findings of this study. For instance, Zhao et al. demonstrated that AI models, particularly those utilizing deep learning algorithms, achieved higher diagnostic accuracy than physicians in detecting STEMI cases from ECG readings [11]. Similarly, research by Günay et al. revealed that GPT-4 outperformed emergency and cardiology specialists in text-based ECG assessments across both routine and complex questions [12]. However, in visual ECG evaluations, cardiologists consistently surpassed GPT-4, GPT-4o, and Gemini, although GPT-4o exhibited superior overall ECG accuracy compared to other AI models [13]. Likewise, Paslı and colleagues emphasized GPT-4′s effective performance in emergency department triage, while noting that models like Gemini displayed tendencies toward over-triage, potentially increasing resource utilization while minimizing the risk of overlooking critical patients [14]. Studies also suggest that ChatGPT, particularly the GPT-4o version, performs on par with inexperienced doctors for straightforward clinical questions but fails to reach gold-standard accuracy in ambiguous or complex cases [15]. Furthermore, Mehnen et al. observed that GPT models require additional guidance in rare diseases or highly complex cases [16]. In another study evaluating ChatGPT’s responses to clinical questions, the model achieved a 90% accuracy rate, indicating a low likelihood of providing incorrect or incomplete information, while suggesting that further parameter optimization could enhance its diagnostic performance [17]. AI tools like ChatGPT, leveraging large datasets and deep learning algorithms, hold considerable promise as decision-support tools comparable to clinical specialists [18]. These technologies, particularly in critical medical emergencies where rapid intervention is vital, can support effective care. However, their integration into clinical practice must be guided by established medical protocols and human oversight to ensure reliability and precision [7,18].

This study stands out as a pioneering effort that compares the responses of clinicians from different specialties, such as emergency medicine and cardiology, with those of artificial intelligence tools using a detailed case set of 20 cardiological emergencies. These cases are designed to closely resemble real-life scenarios and incorporate critical clinical nuances, offering a comprehensive framework for evaluation. The results demonstrate that both cardiologists and ChatGPT provided correct answers to all twenty cardiological case scenarios. However, ChatGPT’s responses were found to be more comprehensive and detailed. In contrast, the responses from emergency physicians showed relatively lower performance in terms of accuracy and detail. AI models like GPT-4o have been shown to perform with high accuracy in common scenarios, such as acute cardiac emergencies, suggesting that its advanced architecture allows for a better understanding of the complexities involved in cardiovascular events. The inclusion of prevalent diseases in the model’s training data further contributes positively to its performance [9]. These findings suggest that ChatGPT holds significant potential as a valuable and complementary tool in clinical decision-making processes, offering speed, scope, and precision alongside human expertise.

Emergency physicians have highlighted their need for consultation in complex cardiological emergencies, such as Brugada Syndrome (Question 5), critical scenarios requiring the reversal of novel oral anticoagulants (Question 8), and STEMI during pregnancy (Question 15). In real-world settings, managing such cases poses significant challenges for emergency physicians. Additionally, the tendency to interpret elevated troponin levels and EKG abnormalities predominantly as acute coronary syndromes often leads to the oversight of non-cardiac causes, such as pulmonary embolism, sepsis, or COPD. In contrast, ChatGPT’s ability to provide guideline-based recommendations offers a distinct advantage in emergency scenarios. Its capacity to consider non-cardiac contributors to troponin elevation and EKG changes enhances differential diagnosis and provides valuable support for clinicians working under time constraints. For example, in hypertensive crisis cases, ChatGPT’s recommendation of rapid intravenous treatment and urgent imaging presents a more effective approach compared to the slower oral therapy commonly preferred by emergency physicians. Similarly, in high-risk acute coronary syndrome cases with ongoing angina, ChatGPT emphasized the importance of early coronary angiography, whereas emergency physicians tended to adopt a more cautious approach by monitoring troponin levels. Furthermore, in a suspected pulmonary embolism case involving chronic kidney disease, ChatGPT’s systematic prioritization of diagnostic tests stood out as a cost-effective and patient-centered strategy. These findings suggest that ChatGPT, by adhering to evidence-based guidelines, has the potential to serve as a valuable decision-support tool in critical care settings. However, its limitations in assessing real-time patient history and clinical context position it not as a replacement for human expertise but as a complementary aid. By improving accuracy, speed, and adherence to clinical guidelines, ChatGPT demonstrates promise as an innovative decision-support mechanism capable of enhancing patient outcomes.

In emergency departments, especially in high-risk cases like cardiovascular emergencies, less experienced emergency physicians may struggle to make decisions with limited information. Challenges such as limited access to cardiology expertise during shifts or busy patient flow can lead to errors in decision-making. In this context, ChatGPT’s ability to quickly access accurate information, provide guideline-consistent recommendations, and deliver detailed responses to complex cases makes it a potential “artificial cardiology consultant”, Such usage could enhance patient safety by preventing errors. Therefore, integrating AI tools into clinical practice, particularly in emergency medicine, offers a significant opportunity to improve patient outcomes.

In our study, ChatGPT provided the fastest responses, followed by cardiologists, while emergency physicians gave the slowest answers. The difference in response times between AI systems and clinicians reflects the distinct nature of their decision-making processes. When clinicians answer a question, they not only rely on analytical thinking and theoretical medical knowledge but also incorporate intuitive and emotional factors. Their responses are informed by previous patient encounters, medical literature, clinical cases shared with colleagues, and the individual characteristics of each patient. Clinicians synthesize multidimensional data, such as a patient’s medical history, current physical findings, living conditions, and social background, to deliver personalized responses. This comprehensive analysis often takes more time than AI’s rapid responses but results in decisions that are more holistic, individualized, and patient-centered. This approach tends to produce more reliable and effective outcomes, especially in complex and unique cases [19]. In contrast, AI systems can quickly access large datasets and process information using advanced algorithms, generating responses within seconds. However, the speed of AI does not always guarantee accuracy, as the model may produce errors when it fails to fully grasp the context. Therefore, the advantage of AI’s speed must be balanced with clinical accuracy and reliability. The best outcomes are achieved when the computational power of AI is combined with the experience and judgment of clinicians.

Although AI systems provide quick access to large datasets and often deliver accurate results, they are prone to producing false or inconsistent information, a phenomenon known as “hallucination”, This occurs due to data limitations, flawed learning processes, or an inadequate understanding of context [20]. Similarly, clinicians can make incorrect decisions due to cognitive biases, distractions, or gaps in knowledge [21]. However, humans can refrain from responding in moments of uncertainty or choose not to answer when they recognize the limits of their knowledge. This ability reflects the concept of “metacognition”, whereby clinicians are aware of and manage their cognitive processes [22]. In contrast, AI systems lack such self-awareness and tend to provide responses to every query, regardless of context [23]. This behavior can increase the risk of errors in critical medical decisions. The inability of AI to recognize its knowledge boundaries poses significant risks, potentially leading to incorrect decisions that may have serious adverse effects on patient health in clinical settings. Therefore, improving the reliability of AI systems requires human oversight and the development of the ability to withhold responses when appropriate, especially in cases of insufficient information.

Focusing solely on the limitations of AI in clinical decision-making can be misleading, as the decision-making processes of human clinicians also carry risks of bias, variability, and error. Achieving a “perfect” clinical decision-making system is undoubtedly an important goal, but waiting for such an ideal may result in unnecessary delays in adopting the benefits of AI. The performance of AI should be evaluated not only against idealized standards but also in comparison with the real-world decision-making processes of clinicians. This approach allows for more achievable and realistic goals. In medical education and professional training, clinicians are expected to meet certain success thresholds in exams as a measure of their clinical competence. Typically, a 60–70% success rate is considered a benchmark for clinical proficiency. If AI systems can achieve an accuracy rate of over 70% in medical questions, this suggests that their theoretical knowledge is approaching that of human clinicians [24]. However, AI has not yet reached the maturity required to replace the experience, knowledge, and skills of professional doctors, particularly in diagnosing and treating medical conditions and building patient-doctor relationships. That said, the continuous improvement capability of AI offers a distinct advantage over the individual development challenges faced by clinicians.

In rural or underdeveloped regions, healthcare systems with limited personnel, materials, and resources often struggle to provide even basic services. In such areas, the lack of sufficient healthcare professionals and restricted access to the most up-to-date medical knowledge can make it difficult to make accurate and timely clinical decisions. Expanding the use of AI tools in medical practice holds significant potential for promoting standardization in healthcare [25]. As it is well known, achieving a uniform approach among physicians is challenging, as clinical decisions tend to vary based on individual experience and level of expertise. In contrast, AI models, trained on large datasets and evidence-based knowledge, can minimize variability and provide more consistent decisions. AI integrates the expertise of multiple professionals, surpassing personal biases, and creating a data-driven, objective “gold standard”, This improves clinical accuracy and has the potential to reduce variability in decision-making among healthcare providers. Providing access to specific AI tools for all clinicians can enhance consistency in diagnostic and treatment processes, leading to significant improvements in patient care quality. Variability in decision-making, especially among healthcare professionals with different levels of clinical experience and expertise, can become more standardized through the use of AI-powered tools. Furthermore, the standardization offered by AI can reduce medical errors, enhance patient safety, and facilitate equitable access to high-quality healthcare for all patients, regardless of geographical or socioeconomic factors [7,25].

The use of AI applications in healthcare brings various challenges. Data sharing, a key component in the development of AI algorithms, raises concerns regarding patient privacy and data security. Algorithmic transparency is essential to ensure that decision-making processes are understandable and traceable; otherwise, patient safety in clinical practice may be compromised. Furthermore, the potential of AI to generate unexpected outcomes highlights the need for regulatory frameworks. The sustainability of AI technologies also requires the effective management of financial resources. Lastly, the risk of unauthorized use must be addressed, and ethical guidelines, limitations, and accreditations should be established by relevant authorities to prevent misuse, protect patient privacy, and safeguard confidentiality. As AI becomes more integrated into healthcare systems, careful management of these challenges will be critical to maximizing potential benefits and minimizing risks. To balance innovation with ethical responsibility, collaboration between policymakers, healthcare professionals, and technology developers will be essential [18,26].

## 5. Limitations

One of the primary limitations of our study is that real-life clinical cases are often more complex, dynamic, and unstructured compared to the scenarios presented in this research. The absence of diagnostic tools, such as physical examinations, electrocardiograms, or chest X-rays, limits the scope of these scenarios and may negatively impact ChatGPT’s performance by restricting its ability to interpret subtle clinical nuances that are critical in emergency settings. Additionally, inexperienced clinicians may face challenges in selecting and accurately communicating critical information to AI systems, which could compromise the accuracy of the recommendations provided and the overall effectiveness of decision-making processes. Moreover, the small sample size of 20 clinical scenarios restricts the generalizability of our findings, as it does not adequately reflect the diversity of real-world medical cases. Another significant limitation is that this study did not evaluate ChatGPT’s impact on patient outcomes, clinician workload, or patient satisfaction, leaving critical real-world implications unexplored. While ChatGPT occasionally demonstrates the ability to identify critical information that clinicians may overlook, it also has a tendency to miss important clinical details [27]. This limitation stems from various factors, including insufficient contextual data, challenges in evaluating negative findings, difficulties in processing unstructured information, and the lack of clinical reasoning capabilities. Since ChatGPT operates solely based on the data provided, incomplete or poorly articulated inputs can lead to the omission of critical insights. Similarly, its capacity to account for negative findings, which are often crucial for differential diagnosis, is limited unless such findings are explicitly stated. Additionally, the interpretation and application of AI-generated recommendations can be influenced by the expertise, cultural perspectives, and potential biases of the clinicians utilizing these tools, which may impact decision-making processes and clinical outcomes.

Considering these limitations, it is evident that ChatGPT is best suited as a complementary decision-support tool rather than a standalone solution. While it holds promise for enhancing accuracy and efficiency in clinical workflows, careful oversight by experienced clinicians remains essential to ensure safe and effective decision-making in patient care.

## 6. Conclusions

This study demonstrates the potential of AI-based tools, like ChatGPT, to assist in clinical decision-making for cardiological emergencies. Despite being designed as a general-purpose chatbot, ChatGPT displayed high accuracy and the ability to deliver fast, comprehensive responses, making it a valuable asset in high-stress environments like emergency departments. The absence of physical exams and imaging studies, while limiting, did not significantly hinder its performance, which is impressive given the context.

While ChatGPT is not yet suited for standalone clinical use, its rapid development indicates that such capabilities may arrive sooner than expected. Integrating AI tools into healthcare could provide crucial support to overburdened clinicians by offering quick access to information and guideline-based recommendations.

Safe implementation, however, requires human oversight and careful management of potential biases in AI models. Thus, AI must complement, rather than replace, clinical judgment. Further research is needed to assess the real-world impact of AI tools on patient outcomes and healthcare delivery. These technologies are rapidly progressing toward becoming integral components of clinical practice.

## Figures and Tables

**Table 1:** Performance comparison of ChatGPT, cardiologists, and emergency physicians in terms of success, accuracy, and completeness.

Group	Participant	Success Rate (%)	Accuracy (Mean ± SD)	Completeness (Mean ± SD)	Test Value
**Emergency** **Physicians (*n* = 3)**	Physician A	75.0	4.18 ± 1.19	1.41 ± 0.51	22.909 ***
	Physician B	65.0	4.06 ± 1.30	1.59 ± 0.79	
	Physician C	80.0	4.53 ± 0.94	1.65 ± 0.61	
	Average	73.3	4.14 ± 1.15	1.64 ± 0.71	
**Cardiologists (*n* = 3)**	Cardiologist A	100.0	5.70 ± 0.47	2.20 ± 0.52	
	Cardiologist B	100.0	5.80 ± 0.52	2.20 ± 0.41	
	Cardiologist C	100.0	5.75 ± 0.44	2.30 ± 0.47	
	Average	100.0	5.75 ± 0.30	2.23 ± 0.36	
**AI System**	ChatGPT-4o	100.0	5.85 ± 0.37	2.75 ± 0.55	

*** Test: Chi-square Fisher exact test.

**Table 2:** Comparison of response times between emergency physicians, cardiologists, and ChatGPT-4o.

Group	Participant	Response Time (Minute)
**Emergency Physicians**	Physician A	33.64
	Physician B	33.14
	Physician C	32.53
	Average	33.1
**Cardiologists**	Cardiologist A	29.12
	Cardiologist B	28.28
	Cardiologist C	27.55
	Average	28.32
**AI System**	ChatGPT-4o	11.27

**Table 3:** Comparison of accuracy and completeness scores between ChatGPT, cardiologists, and emergency physicians.

Raters		Mean	SD.	Mean Rank	Min.	Max.	Test	Post Hoc
**Accuracy**	ChatGPT 4.o ^1^	5.85	0.37	40.80	5.00	6.00	33.802 ***	2 < 1 *** 2 < 3 ***
	Emergency Physician (Avg.) ^2^	4.14	1.15	11.83	1.00	6.00		
	Cardiologist (Avg.) ^3^	5.75	0.30	34.10	5.00	6.00		
**Completness**	ChatGPT 4.o ^1^	2.75	0.55	42.95	1.00	3.00	27.671 ***	2 < 1 ** 2 < 3 ** 1 > 3 *
	Emergency Physician (Avg.) ^2^	1.64	0.71	14.86	1.00	4.00		
	Cardiologist (Avg.) ^3^	2.23	0.36	29.23	1.67	3.00		

Test: Kruskal–Wallis H test, Post Hoc: Mann–Whitney U test with Bonferroni correction, *** *p* < 0.001, ** *p* < 0.01, * *p* < 0.05. ^1^: ChatGPT-4.0 results, ^2^: Emergency physicians (average of individual scores), ^3^: Cardiologists (average of individual scores).

**Table 4:** Statistical analysis of accuracy and completeness Scores for ChatGPT, cardiologists, and emergency physicians.

Comparison	Mean Rank (ChatGPT vs. Comparator)	*p*-Value
**Accuracy**		
ChatGPT-4o vs. Emergency Physician	5.85 vs. 4.14	<0.001
ChatGPT-4o vs. Cardiologist	5.85 vs. 5.75	<0.001
Emergency Physician vs. Cardiologist	4.14 vs. 5.75	0.184
**Completeness**		
ChatGPT-4o vs. Emergency Physician	2.75 vs. 1.64	<0.001
ChatGPT-4o vs. Cardiologist	2.75 vs. 2.23	0.008
Emergency Physician vs. Cardiologist	1.64 vs. 2.23	0.021

## Data Availability

The datasets generated and analyzed during this study are available from the corresponding author, Muhammet Geneş, upon reasonable request.

## Supplementary Materials

The following supporting information can be downloaded at: https://www.mdpi.com/article/10.3390/diagnostics14232731/s1. Supplementary Materials.
