# Supplementary material for: A Clinical Evaluation of Cardiovascular Emergencies: A Comparison of Responses from ChatGPT, Emergency Physicians, and Cardiologists
Source: Diagnostics (Basel). 2024 Dec 4;14(23):2731. doi: 10.3390/diagnostics14232731 (PMC11639855; doi:10.3390/diagnostics14232731)
Supplement: Supplementary file 1 [file diagnostics-14-02731-s001.zip › diagnostics-3309373-supplementary.pdf]

Supplementary Materials
Responses Provided by ChatGPT to Hypothetical Clinical Scenarios

Q1) A 58-year-old female patient with a history of hypertension and high cholesterol presents to the emergency department with sudden symptoms of chest pressure, shortness of breath, and cold sweats. Her blood pressure is 160/95 mmHg, and her heart rate is 90 beats per minute. On physical examination, lung sounds are normal, and no heart murmur is detected. The ECG shows sinus rhythm with a prominent R wave in lead V2, mild ST segment elevation (approximately 0.5 mm) in lead III, and horizontal ST segment depression (approximately 2 mm) in leads V1–V3.
What is the most appropriate diagnostic approach, and what treatment plan should be implemented for this patient?

A1) In this case, a 58-year-old woman with a history of hypertension and high cholesterol is suspected of having an acute coronary syndrome (ACS). Symptoms such as chest pressure, shortness of breath, and cold sweats are typically indicative of myocardial ischemia.

ECG findings show ST-segment elevation in lead III and ST-segment depression in leads V1–V3. This situation suggests acute coronary syndrome, particularly raising the possibility of non-ST-segment elevation myocardial infarction (NSTEMI).

Diagnostic Approach:

- **Troponin:** Troponin levels should be checked to support the diagnosis of acute coronary syndrome. Elevated troponin levels indicate myocardial damage.
- **Blood Tests:** Other biochemical parameters should be evaluated (electrolytes, kidney function, complete blood count).
- **Echocardiography:** It may be performed to assess regional wall motion abnormalities and myocardial function.
- **Coronary Angiography:** This may be needed to confirm ACS and identify stenosis in the coronary arteries.

Treatment Plan:

- **Aspirin:** Antiplatelet therapy should be administered immediately (160–325 mg, chewed).
- **Nitroglycerin:** Sublingual nitroglycerin can be used to relieve pain and dilate the coronary arteries (if the blood pressure is stable and there are no contraindications).
- **Heparin or low molecular weight heparin (LMWH):** Anticoagulation therapy should be initiated.
- **Beta-Blocker:** If the patient's blood pressure is stable and there are no contraindications, a beta-blocker (such as metoprolol) can be started.
- **Statin:** High-dose statins (e.g., atorvastatin) should be given to control lipid levels.
- **Urgent Coronary Angiography and PCI:** If symptoms persist or if high-risk features are present, the patient should be urgently referred for percutaneous coronary intervention (PCI).

This approach aims to control the patient's symptoms and prevent potential complications.

Q2) A 63-year-old male patient with no known medical history presents to the emergency department with complaints of severe headache and dizziness. He reports no chest pain or shortness of breath. He is not on any medication. His blood pressure is 185/100 mmHg, and his heart rate is 55 beats per minute and regular. The ECG shows T wave inversions in the precordial leads.
What is the most appropriate next step in management for this patient?

A2) In this case, the 63-year-old male patient's presentation with severe headache and dizziness suggests a possible hypertensive crisis or hypertensive encephalopathy.
Key points for diagnosis and assessment:

- **Blood Pressure:** 185/100 mm Hg, indicating a high-risk hypertensive crisis that requires emergency treatment.
- **Heart Rate:** 55 bpm, indicating bradycardia, which should be considered in this hypertensive setting. Bradycardia could be related to increased intracranial pressure or other serious cardiac conditions.
- **ECG Findings:** T wave inversions in the precordial leads may suggest ischemic changes. This could indicate myocardial ischemia related to the hypertensive crisis.

Next steps in management:

1. **Brain CT or MRI:** Due to the severe headache and dizziness, brain imaging is required to rule out intracranial hemorrhage or cerebrovascular events.
2. **Antihypertensive Treatment:**
    - Intravenous medications are preferred to lower blood pressure rapidly but in a controlled manner. First-line agents include **labetalol, nitroprusside, or esmolol**.
    - However, it is crucial to avoid lowering the blood pressure too quickly, as this could reduce cerebral perfusion.
3. **Electrolytes and Biochemistry:** Renal function and electrolyte imbalances should be evaluated, as they are important in the management of hypertensive crises.
4. **ECG Monitoring:** Repeating the ECG to monitor for myocardial ischemia or any progression of ischemic changes will be helpful.

In conclusion: The patient should be managed for a hypertensive crisis, with brain imaging performed, and blood pressure carefully controlled. Although the absence of chest pain makes an acute coronary syndrome less likely, the ischemic changes (T wave inversions) may still indicate myocardial stress.

Q3) A 55-year-old female patient with a history of chronic kidney disease presents to the emergency department with sudden-onset shortness of breath and sharp right-sided chest pain. She recently returned from a long flight. On physical examination, she is tachypneic, tachycardic, and her blood pressure is normal. Due to chronically elevated serum creatinine levels, a chest CT angiography cannot be performed.
What is the most appropriate diagnostic approach to exclude pulmonary embolism in this patient?

A3) This patient presents with sudden onset shortness of breath and right-sided chest pain following a long flight, placing her at high risk for pulmonary embolism (PE). The history of chronic kidney disease complicates the use of contrast agents in diagnostic tests, so alternative diagnostic approaches should be considered.
Most Appropriate Diagnostic Approach:

1. **D-Dimer Test:**
    - A simple and rapid test that can be used initially to assess the suspicion of PE. A negative D-dimer result may rule out PE. However, given the patient's high risk, a negative result may not completely exclude PE.
2. **Ventilation-Perfusion (V/Q) Scan:**
    - A preferred imaging modality in patients with kidney disease, as it does not require contrast. V/Q mismatch may indicate the presence of PE. Since a chest CT angiography cannot be performed, this is a suitable alternative for this patient.
3. **Transthoracic Echocardiography (TTE):**
    - Particularly useful in cases of hemodynamic instability or high-risk PE to detect indirect signs of PE, such as acute right ventricular dilation. However, TTE alone is not sufficient to definitively diagnose PE.
4. **Lower Extremity Doppler Ultrasound:**
    - Since the patient has a history of a long flight, deep vein thrombosis (DVT) in the legs is a possibility. Doppler ultrasound can be used to evaluate for thrombosis in the leg veins. The detection of DVT would support the diagnosis of PE.

Conclusion: The most appropriate diagnostic approach for this patient includes performing a D-dimer test, followed by a V/Q scan. Additionally, echocardiography can be used to assess right ventricular function, and lower extremity Doppler ultrasound may help confirm PE. It is important to establish the diagnosis before starting treatment, but if the patient is hemodynamically unstable, treatment should not be delayed.

Q4) A 66-year-old male patient with chronic obstructive pulmonary disease (COPD) presents to the emergency department with complaints of shortness of breath and cough. His respiratory rate is 27 breaths per minute, and his oxygen saturation is 89%. Arterial blood gas analysis reveals low partial pressure of oxygen (PaO_2 54 mmHg) and carbon dioxide retention ($PaCO_2$ 62 mmHg). The ECG shows normal sinus rhythm with no significant ischemic findings. Laboratory results indicate an elevated troponin level of 42 ng/L (reference range 0-35 ng/L), while creatinine levels are within normal limits.
What is the most appropriate management strategy for this patient?

A4) In this case, we are dealing with a patient experiencing an exacerbation of COPD (chronic obstructive pulmonary disease). The clinical findings include low oxygen saturation (89%) and arterial blood gas results showing hypoxemia (PaO_2: 54 mmHg) and hypercapnia ($PaCO_2$: 62 mmHg). Additionally, there is an elevated troponin level (42 ng/L), which may indicate myocardial stress.

Optimal Management Strategy:

1. **Oxygen Therapy:**
    - Oxygen therapy should be initiated as the patient’s oxygen saturation is 89%. However, caution is necessary with oxygen therapy in COPD patients, as excessive oxygen can worsen hypercapnia. The target oxygen saturation should be maintained between 88-92%.
    - High-flow nasal cannula or a venturi mask can be used as options for oxygen therapy.
2. **Noninvasive Mechanical Ventilation (NIMV):**
    - Due to elevated $PaCO_2$ (hypercapnia) and respiratory distress, NIMV is indicated. This is one of the first-line treatment options in COPD exacerbations and helps relieve the workload on the respiratory muscles.

3. **Bronchodilator and Corticosteroid Therapy:**
   - Inhaled bronchodilators (e.g., short-acting beta-agonists and anticholinergics) should be used to treat the acute exacerbation.
   - Systemic corticosteroids (e.g., prednisone) may be given to reduce the exacerbation. These are typically used for a short period (5-7 days).
4. **Antibiotic Therapy (If Indicated):**
   - If there are signs of infection (fever, purulent sputum), antibiotic therapy should be initiated. Common pathogens in COPD exacerbations include *Streptococcus pneumoniae* and *Haemophilus influenzae*.
5. **Elevated Troponin:**
   - The rise in troponin levels may indicate myocardial injury. Rather than acute coronary syndrome, this is likely related to "type 2 myocardial infarction," which results from an imbalance between oxygen demand and supply. The patient's hemodynamic and cardiac status should be closely monitored.
6. **Fluid Management:**
   - Careful attention to fluid balance is crucial, as excessive fluid administration can lead to pulmonary edema in COPD patients. Isotonic fluids should be given cautiously.

Conclusion: Noninvasive mechanical ventilation, careful oxygen therapy, bronchodilators, corticosteroids, and antibiotics (if infection is present) are recommended for this patient. The elevated troponin levels may reflect myocardial stress, so cardiac monitoring is advised.

Q5) A 25-year-old male patient presents to the emergency department after experiencing a syncopal episode during breakfast. He appears very anxious and reports that his brother recently died suddenly. The ECG shows ST-segment elevation in V1-V2 leads and a right bundle branch block.
What is the next most appropriate step in management?

A5) In this case, a 25-year-old male patient presents with a complaint of syncope and the ECG findings show ST-segment elevation in leads V1-V2 and right bundle branch block. Additionally, the patient has a family history of sudden death. These findings are highly suggestive of **Brugada syndrome**.
Brugada syndrome is a genetic ion channel disorder that can lead to sudden cardiac death in young individuals. It is characterized by ST-segment elevation and right bundle branch block. Patients with this condition are at risk of developing ventricular arrhythmias.
The next most appropriate step in management:

1. **Cardiology Consultation:**
   - Brugada syndrome carries a risk of sudden cardiac death, so an urgent cardiology consultation is necessary.
2. **Electrophysiological Study and ICD (Implantable Cardioverter Defibrillator):**
   - This patient is at risk of sudden death, especially with high-risk factors such as cardiac arrest or syncope. ICD implantation is recommended. An electrophysiological study can be performed to provoke ventricular arrhythmias and assess the degree of risk.
3. **Family Screening:**
   - Since Brugada syndrome is a genetic condition, screening of the patient's family members may be necessary. The sudden death of the patient's sibling suggests a possible genetic predisposition.
4. **Urgent Treatment and Monitoring:**

  - The patient should be monitored with continuous ECG to detect the development of ventricular arrhythmias. If the patient develops ventricular fibrillation, immediate defibrillation will be required.
  - The patient should be advised to avoid medications that can trigger arrhythmias (e.g., sodium channel blockers) and to avoid situations that may induce fever, as fever can increase the risk of arrhythmias in Brugada syndrome.

Conclusion: The most appropriate approach for this patient involves urgent cardiology consultation, ICD implantation, and an electrophysiological study. Close monitoring for arrhythmia risk is essential.

Q6) An 88-year-old male patient with end-stage COPD presents to the emergency department with severe shortness of breath and narrow complex tachycardia. Intravenous adenosine was administered to stop the tachycardia, but it was ineffective. Based on the patient's medical history, what should be the next most appropriate step in treatment?

A6) In this case, an 88-year-old patient with end-stage COPD presents with severe shortness of breath and narrow complex tachycardia. Intravenous adenosine was administered, but the tachycardia did not resolve. The following points should be considered in determining the next best step in treatment:

1. Cause of Narrow Complex Tachycardia:

- Narrow complex tachycardia is often associated with supraventricular tachycardia (SVT). Adenosine is effective in AV nodal re-entry tachycardias, but it was not effective in this case.
- In COPD patients, atrial fibrillation or atrial flutter is also common and can lead to supraventricular tachycardia.

2. Next Treatment Step:

- **Beta-Blockers or Calcium Channel Blockers:** If adenosine is ineffective, beta-blockers (e.g., metoprolol) or nondihydropyridine calcium channel blockers (e.g., diltiazem or verapamil) may be considered for rate control. However, beta-blockers should be used cautiously in COPD patients as they may increase the risk of bronchospasm. Therefore, calcium channel blockers may be a safer option.
- **Amiodarone:** If atrial fibrillation or flutter is suspected, antiarrhythmic drugs like amiodarone can be considered. Amiodarone is effective in both rate control and rhythm conversion and is generally preferred in COPD patients.

3. Electrical Cardioversion:

- If the patient becomes hemodynamically unstable or if rapid resolution of the tachycardia is needed, electrical cardioversion should be considered. This may provide a quick solution, especially if the patient’s condition worsens.

4. COPD Management:

- In addition to treating the tachycardia, the COPD exacerbation should be managed aggressively. Bronchodilators (e.g., nebulized albuterol and ipratropium), systemic corticosteroids, and, if necessary, noninvasive mechanical ventilation (NIMV) should be utilized. Oxygen therapy should be administered carefully, considering the risk of hypercapnia.

Conclusion: In this patient with adenosine-resistant narrow complex tachycardia, calcium channel blockers (e.g., diltiazem) or amiodarone should be considered. If the patient becomes hemodynamically unstable, urgent cardioversion may be necessary. The COPD exacerbation should also be appropriately treated.

Q7) A 27-year-old male with no known chronic conditions presents to the emergency department with narrow complex tachycardia. His vital signs are as follows: blood pressure 112/72 mmHg, heart rate 175 bpm, body temperature 36.7°C, and room air SpO2 at 97%. Upon further questioning, the patient reports excessive alcohol consumption over the weekend. The ECG shows an irregularly irregular rhythm at around 170 bpm. What is the most appropriate management strategy for this patient?

A7) In this case, a 27-year-old male presents with narrow complex tachycardia. The presence of an irregularly irregular rhythm on ECG suggests atrial fibrillation (AF). The patient also has a history of excessive alcohol consumption over the weekend, which points to "Holiday Heart Syndrome," a transient atrial fibrillation triggered by alcohol intake.

Optimal Management Strategy:

1. **Assessment of Hemodynamic Stability:**
   - The patient's blood pressure (112/72 mmHg) and oxygen saturation (97%) are stable, indicating no signs of hemodynamic instability that would require urgent cardioversion. Therefore, rate control and rhythm control strategies can be considered.
2. **Rate Control:**
   - The first step in treatment is controlling the ventricular rate. Beta-blockers (e.g., metoprolol) or calcium channel blockers (e.g., diltiazem or verapamil) can be used. These medications help slow the ventricular rate and alleviate symptoms.
3. **Rhythm Control:**
   - Atrial fibrillation induced by alcohol intake usually resolves on its own within 48 hours. However, if symptoms persist or AF does not resolve spontaneously, pharmacological cardioversion can be considered. Antiarrhythmic medications such as amiodarone, flecainide, or propafenone can be used.
   - Rhythm control with medications is generally preferred if there is no underlying structural heart disease. Since AF after excessive alcohol intake is typically transient, rhythm control may not be the first choice.
4. **Treatment of Underlying Cause:**
   - The patient should be advised to reduce alcohol consumption, as alcohol increases atrial irritability and can trigger arrhythmias like AF.
5. **Anticoagulation:**
   - Since the patient's AF duration is less than 48 hours and there are no additional risk factors, urgent anticoagulation is generally not required. However, if AF persists longer, anticoagulation should be considered based on the CHA2DS2-VASc score.

Conclusion: For this patient, rate control should be achieved initially with a beta-blocker or calcium channel blocker. If symptoms persist or AF does not resolve, pharmacological cardioversion can be performed. Anticoagulation should be evaluated based on the duration of AF and the patient's risk factors.

Q8) A 66-year-old male with a history of atrial fibrillation on dabigatran 150 mg twice daily for stroke prevention presents to the emergency department with massive gastrointestinal bleeding. Upon arrival, he is unconscious, intubated, and hypotensive. Evaluation reveals a

life-threatening hemorrhage requiring emergency surgery. What is the most appropriate treatment approach for reversing anticoagulation in this patient?

A8) In this case, a 66-year-old patient is taking dabigatran for atrial fibrillation and presents with massive gastrointestinal bleeding. The patient is unconscious, intubated, and hypotensive, indicating a life-threatening emergency with active bleeding. Immediate reversal of dabigatran's anticoagulant effect is required.

Reversal of Dabigatran Anticoagulation:

1. **Idarucizumab (Praxbind):**
   - Idarucizumab is the specific antidote for dabigatran and is used to rapidly reverse its anticoagulant effects. Idarucizumab can halt dabigatran's action within minutes, helping to control bleeding.
   - **Dosage:** Idarucizumab is administered as two separate intravenous boluses (2.5 grams each) for a total dose of 5 grams.
2. **Supportive Treatment:**
   - **Fluid Resuscitation:** Since the patient is hypotensive, rapid fluid replacement should be initiated. Isotonic fluids and, if necessary, blood transfusions may be required.
   - **Blood Product Transfusion:** In patients with severe bleeding, packed red blood cells should be given to maintain hemoglobin levels and blood volume. Fresh frozen plasma (FFP) can also be administered if needed.
   - **Surgical Intervention:** In this case, the source of bleeding should be promptly addressed with surgical intervention to achieve hemostasis.
3. **Alternative Therapies:**
   - If idarucizumab is unavailable or does not provide adequate response, **hemodialysis** may be considered as an alternative method to remove dabigatran from the blood, as dabigatran is dialyzable.
   - **Activated charcoal** may be administered if dabigatran ingestion occurred within the last few hours to limit further absorption.

Conclusion: The optimal treatment approach for this patient involves rapid reversal of dabigatran with idarucizumab, fluid and blood transfusions to stabilize hemodynamics, and surgical intervention to control the source of bleeding.

Q9) A 75-year-old male with a known history of atrial fibrillation who is regularly taking rivaroxaban presents to the emergency department with severe chest pain. The ECG shows an acute ST-elevation myocardial infarction (STEMI). The patient is being referred to the cardiology team for urgent percutaneous coronary intervention (PCI). Considering that the patient is on rivaroxaban, how should the P2Y12 inhibitor and heparin loading dose be managed?

A9) In this case, a 75-year-old male patient with a history of atrial fibrillation is on regular rivaroxaban and presents to the emergency department with an acute ST-elevation myocardial infarction (STEMI). The patient is being referred for urgent percutaneous coronary intervention (PCI). Oral anticoagulants like rivaroxaban present challenges in the management of anticoagulation and antiplatelet therapy, so a careful approach is required.

Management Plan:

1. **Heparin Loading Dose:**

- In a patient taking rivaroxaban, additional anticoagulation is needed before PCI. However, since the anticoagulant effect of rivaroxaban is still present, the heparin loading dose should be adjusted carefully.
- **Dose:** The standard heparin dose is 70-100 U/kg, but due to the ongoing rivaroxaban effect, a lower dose may be used. Typically, around 50-60 U/kg of heparin is recommended. This ensures adequate anticoagulation in combination with rivaroxaban's effect.

2. **Choice of P2Y12 Inhibitor:**
    - Since rivaroxaban therapy is ongoing, dual antiplatelet therapy with a P2Y12 inhibitor is required. However, given the bleeding risk, careful selection is important.
    - **Ticagrelor** or **prasugrel** can be considered in this situation. Prasugrel, in particular, should be used cautiously in patients taking rivaroxaban due to the higher bleeding risk. Ticagrelor is generally considered a safer option.
    - **Dose:** A loading dose of 180 mg ticagrelor should be administered.
3. **Assessment of Bleeding Risk:**
    - The combination of an anticoagulant (rivaroxaban) and antiplatelet therapy increases the risk of bleeding. Therefore, therapy must be carefully monitored based on the patient's bleeding risk. If the bleeding risk is high, lower doses of anticoagulants and antiplatelet agents may be considered.
4. **Continuation of Rivaroxaban:**
    - If the patient continues on long-term anticoagulation after PCI, the risks and benefits of combined antiplatelet and anticoagulant therapy must be evaluated. **Triple therapy** (aspirin, P2Y12 inhibitor, and anticoagulant) may be used for a short period, after which one antiplatelet agent may be discontinued.

Conclusion: The heparin loading dose for this patient should be adjusted considering the effect of rivaroxaban (approximately 50-60 U/kg of heparin). Ticagrelor should be chosen as the P2Y12 inhibitor (180 mg loading dose), and bleeding risk should be closely monitored.

Q10) A 38-year-old male is brought to the emergency department after being stabbed in the chest during a street altercation. What is the appropriate approach for managing a penetrating cardiac trauma?

A10) In this case, a 38-year-old male patient presents to the emergency department after sustaining a stab wound to the chest. This is considered a penetrating cardiac trauma, requiring a rapid and organized intervention. Cardiac injuries can quickly become life-threatening, making emergency management critical.

Management of Penetrating Cardiac Trauma:

1. **Assessment of Patient Stability:**
    - **Hemodynamic Evaluation:** The first step is to quickly assess whether the patient is hemodynamically stable. Hypotension, tachycardia, or signs of shock may indicate severe blood loss or cardiac tamponade.
2. **Rapid Assessment:**
    - **Physical Examination:** The location, size, and depth of the wound should be evaluated. In patients with a chest stab wound, physical signs of cardiac tamponade (hypotension, pulsus paradoxus, venous distension) should be assessed quickly.

- **FAST (Focused Assessment with Sonography for Trauma):** FAST can be used to rapidly detect the presence of fluid (blood) in the chest cavity or pericardium.

3. **Emergency Intervention:**
   - **Pericardiocentesis:** If signs of cardiac tamponade are present and the patient is hemodynamically unstable, emergency pericardiocentesis may be performed. This procedure drains the blood around the heart, relieving pressure and restoring cardiac function.
   - **Emergency Thoracotomy:** If the patient is not hemodynamically stable and there is suspicion of penetrating cardiac injury, an emergency thoracotomy may be required. This procedure can be performed in the emergency department to save the patient's life and directly repair cardiac injuries. Mortality is very high if surgical intervention is not performed in time.
4. **Surgical Consultation:**
   - If the patient becomes stabilized, more advanced surgical interventions should be planned by a cardiothoracic surgeon. The patient may need to be taken to the operating room urgently for cardiac repair or treatment of other traumatic injuries.
5. **Fluid and Blood Product Resuscitation:**
   - **Intravenous Fluids and Blood Transfusion:** In cases of severe blood loss, rapid fluid resuscitation and transfusion of blood products should be initiated. These measures should be continued until hemodynamic stability is achieved.
6. **Imaging and Monitoring:**
   - **Chest X-ray and Echocardiography:** If time and patient stability permit, chest X-rays and echocardiography can provide more information about the extent and location of cardiac injuries.

Conclusion: In cases of penetrating cardiac trauma, emergency pericardiocentesis or thoracotomy may be required. Rapid hemodynamic assessment and intervention are critical. Surgical consultation and advanced imaging should follow to manage life-threatening conditions.

Q11) A 28-year-old male presents to the emergency department with severe chest pain and shortness of breath following cocaine use. His medical history reveals intermittent cocaine use over several years, alcohol dependence, and smoking. He has no other known cardiac risk factors. What is the most appropriate approach for managing this patient? How does cocaine use affect the cardiovascular system?

A11) In this case, a 28-year-old male presents with severe chest pain and shortness of breath following cocaine use. Cocaine has significant effects on the cardiovascular system, particularly leading to myocardial ischemia. The patient also has risk factors such as smoking and alcohol dependency but no other known cardiac risk factors.

Cardiovascular Effects of Cocaine:

1. **Coronary Vasoconstriction:**
   - Cocaine induces strong sympathomimetic effects, causing spasm and vasoconstriction of the coronary arteries. This can lead to myocardial ischemia and even myocardial infarction.
2. **Platelet Activation and Increased Risk of Thrombosis:**
   - Cocaine increases platelet activity, raising the risk of thrombosis. Cocaine users are at risk of coronary artery thrombosis, which can result in myocardial infarction.

3. **Tachycardia and Hypertension:**
   - Cocaine stimulates the sympathetic nervous system, increasing heart rate and blood pressure. Chronic use leads to hypertension, increasing the workload on the heart and causing ischemic damage.
4. **Ventricular Arrhythmias:**
   - Cocaine use can trigger ventricular arrhythmias and sudden cardiac death by disrupting cardiac repolarization, leading to dangerous rhythm disturbances.

Appropriate Management for This Patient:

1. **Emergency Management of Myocardial Ischemia:**
   - Given the patient's severe chest pain and shortness of breath, myocardial ischemia or acute coronary syndrome (ACS) should be suspected.
   - **Nitroglycerin:** This should be administered to alleviate coronary vasoconstriction. Nitroglycerin is effective in relieving the coronary vasospasm caused by cocaine.
   - **Aspirin:** If ACS is suspected, aspirin should be given as an antiplatelet therapy.
2. **Benzodiazepines:**
   - To mitigate the excessive sympathetic stimulation from cocaine, benzodiazepines (such as diazepam or lorazepam) should be administered. These help control anxiety and reduce heart rate and blood pressure.
3. **Avoid Beta-Blockers:**
   - Beta-blockers should not be used in patients who have used cocaine, as they can lead to unopposed alpha receptor activation, worsening coronary vasoconstriction. Instead, nitrates and benzodiazepines should be used.
4. **ECG and Cardiac Enzymes:**
   - An ECG should be performed to evaluate for ischemic changes. Cardiac enzymes such as troponin and CK-MB should be measured to assess myocardial damage.
5. **High-Flow Oxygen Support:**
   - If hypoxemia develops, oxygen therapy should be provided. However, oxygen saturation often remains normal after cocaine use.
6. **Other Supportive Treatment:**
   - If the patient is hypertensive, antihypertensive treatment should be initiated cautiously. The patient should also receive counseling for alcohol and smoking cessation.

Conclusion: Cocaine significantly affects the cardiovascular system, leading to coronary vasospasm, thrombosis, myocardial ischemia, hypertension, and arrhythmias. This patient should be treated with nitroglycerin, aspirin, benzodiazepines, and oxygen support, while beta-blockers should be avoided. ECG and cardiac enzymes should be used to assess ischemic damage.

Q12) A 25-year-old male, unrestrained in a head-on motor vehicle collision, is brought to the trauma bay of the emergency department. The patient is unconscious, with multiple lacerations on his head, a large contusion on the anterior chest, and abdominal distention. What is the appropriate approach for the acute evaluation of a patient with blunt cardiac trauma?

A12) In this case, we are dealing with an unconscious patient involved in a head-on motor vehicle collision. The patient has chest contusions and abdominal distention, which raises concern for blunt cardiac trauma and other traumatic injuries that require careful evaluation.

Acute Assessment of Blunt Cardiac Trauma:

1. **ABC Principles (Airway, Breathing, Circulation):**
   - **Airway:** Since the patient is unconscious, the airway should be assessed immediately. Intubation may be required to secure the airway.
   - **Breathing:** Evaluate the patient's respiratory status. If chest contusions are present, complications such as lung injury (pulmonary contusion) or hemopneumothorax should be considered. Oxygen saturation should be monitored, and a chest tube may be needed if necessary.
   - **Circulation:** Blood pressure, pulse, and peripheral perfusion should be assessed. Hypotension and tachycardia may indicate internal bleeding or cardiac injury.
2. **Echocardiography (FAST):**
   - **Transthoracic Echocardiography (TTE):** This is one of the first imaging modalities to evaluate for cardiac injuries such as tamponade, ventricular rupture, or atrial tears.
   - **FAST (Focused Assessment with Sonography for Trauma):** Performed in the emergency department to identify pericardial or intra-abdominal fluid accumulation (hemorrhage).
3. **Chest X-ray:**
   - A chest X-ray should be done promptly to assess the effects of blunt trauma. Findings such as rib fractures, pleural effusion, hemothorax, or pneumothorax should be evaluated.
4. **Cardiac Enzymes:**
   - In cases of blunt cardiac trauma, measuring cardiac enzymes (troponin, CK-MB) is important to assess myocardial damage. Elevated levels may indicate cardiac contusion.
5. **Electrocardiogram (ECG):**
   - An ECG should be performed to detect arrhythmias, ST-T changes, or conduction blocks, which could be indicative of blunt cardiac trauma.
6. **CT (Computed Tomography):**
   - If the patient is stable, contrast-enhanced CT scans of the chest and abdomen should be obtained. These scans provide detailed information about injuries to the heart, chest, and abdominal organs. Serious findings such as cardiac rupture, aortic dissection, or other vascular injuries can be identified.
7. **Laboratory Tests:**
   - Laboratory tests such as blood gas analysis, complete blood count, electrolytes, coagulation parameters, and lactate levels can provide information about the patient's overall status and the possibility of internal bleeding.
8. **Surgical Consultation:**
   - Abdominal distention and hypotension suggest possible internal bleeding, which may require emergency surgical intervention. If the patient is hemodynamically unstable, they should be referred for surgery immediately.

Conclusion: In a patient with blunt cardiac trauma, initial assessment should follow the ABC principles (airway, breathing, circulation). Immediate echocardiography, FAST, ECG, cardiac enzyme tests, and chest X-ray should be performed, and if necessary, thoracic and abdominal CT scans should be ordered. If internal bleeding or cardiac injuries are detected, surgical intervention may be required.

Q13) A 52-year-old male patient was brought to the emergency department by ambulance after a sudden loss of consciousness at home. When the ambulance crew arrived, the patient had no detectable pulse, and he was determined to be in cardiac arrest. Cardiopulmonary resuscitation (CPR) was initiated immediately, and after 12 minutes, spontaneous blood pressure and pulse were obtained. Upon arrival at the emergency department, the patient remained unconscious, with a palpable pulse and a blood pressure of 90/60 mmHg. There is no known medical history, but the family reports that the patient occasionally complained of chest pain. Physical examination did not reveal any obvious abnormalities. An electrocardiogram (ECG) performed in the emergency department showed no ST elevation. The patient has been stabilized after resuscitation. What would be the most appropriate approach for managing this patient?

A13) In this case, a 52-year-old male patient has been stabilized after resuscitation following sudden loss of consciousness and cardiac arrest. While there is no known history of chronic illness, the patient's prior occasional complaints of chest pain suggest a possible cardiac etiology. However, the absence of ST elevation on the ECG excludes an acute ST-elevation myocardial infarction (STEMI). Therefore, the post-arrest management requires a comprehensive and cautious evaluation.

Optimal Approach:

1. Patient Stabilization and Basic Care:
    - The first step is to maintain hemodynamic stability. The patient's blood pressure is 90/60 mmHg, indicating a tendency towards hypotension, so fluid resuscitation and, if necessary, inotropic agents (e.g., dobutamine) should be considered.
    - To secure the airway and ventilation, the patient should be intubated, and oxygen saturation should be maintained above 94%.
2. Investigation of Arrest Etiology:
    - Cardiac Causes: The history of chest pain coupled with sudden loss of consciousness raises suspicion of a cardiac cause. The absence of ST elevation on the ECG does not rule out acute coronary syndrome. The patient could have had a non-ST elevation myocardial infarction (NSTEMI) or an arrhythmia-induced cardiac arrest.
        - Troponin levels and other cardiac biomarkers should be evaluated to assess myocardial damage.
        - Echocardiography can be used to assess structural abnormalities of the heart, regional wall motion abnormalities, or right ventricular dysfunction.
    - Other Potential Causes:
        - Pulmonary Embolism (PE): The absence of ST elevation on the ECG post-arrest may raise suspicion of a pulmonary embolism. If clinically suspected, a D-dimer test and thoracic CT angiography should be performed.
        - Electrolyte Imbalances: Imbalances in potassium, magnesium, and other electrolytes could have triggered an arrhythmia. A complete blood count, electrolyte levels, and renal function tests should be conducted.
        - Hypo-/Hyperthermia: The patient's body temperature should be monitored, and normothermia should be maintained if needed.

        - Hypoxia: Other causes of cardiac arrest, such as severe hypoxia or CO2 retention, should be investigated.
3. Coronary Angiography Decision:
    - In a patient who has suffered cardiac arrest without ST elevation on the ECG, routine coronary angiography is not indicated. However, given the history of chest pain and the possibility of a cardiac cause, coronary angiography may be considered.
    - Especially if there is elevated troponin or echocardiographic evidence of myocardial damage, coronary artery evaluation will be necessary.
4. Neurological Assessment and Targeted Temperature Management (TTM):
    - One of the key components of post-arrest care in an unconscious patient is ensuring neurological recovery. Targeted temperature management is recommended. This treatment aims to minimize ischemic damage to the brain by controlling the patient's body temperature within the range of 32-36°C.
    - Neurological status should be closely monitored, and brain imaging (e.g., brain CT/MRI) may be required to assess the post-arrest neurological prognosis.
5. Monitoring and Follow-Up:
    - The patient should be admitted to the intensive care unit, with continuous ECG monitoring and close observation of hemodynamic status.
    - Laboratory results, cardiac function, electrolyte balance, and fluid status should be frequently evaluated.

Conclusion: For this patient with no ST elevation on ECG and an unknown arrest etiology, the first priority is to ensure hemodynamic stability, investigate both cardiac and non-cardiac causes, initiate targeted temperature management, and consider coronary angiography if warranted.

Q14) A 76-year-old male presents to the emergency department with complaints of shortness of breath and pleuritic chest pain. His medical history includes diabetes and hypertension. There is a clinical suspicion of pulmonary embolism (PE). His blood pressure is 122/72 mmHg, heart rate is 118 bpm, and oxygen saturation is 89% on 3 L/min nasal cannula. A chest X-ray shows hyperinflation of the lung fields. Troponin levels are elevated at 37 ng/L, and eGFR is 62 mL/min. Computed tomography pulmonary angiography (CTPA) reveals bilateral proximal pulmonary emboli. What is the most appropriate approach and initial treatment for this patient?

A14) In this case, a 76-year-old male presents with shortness of breath and pleuritic chest pain. Pulmonary embolism (PE) was confirmed by computed tomography pulmonary angiography (CTPA), showing bilateral proximal pulmonary emboli. Additionally, elevated troponin levels (37 ng/L) and an eGFR of 62 mL/min provide insight into the patient's renal function and myocardial stress.

Appropriate Approach and Initial Treatment:

1. **Risk Stratification:**
    - The patient has stable vital signs (blood pressure 122/72 mmHg), with no hypotension or shock, indicating non-massive pulmonary embolism. However, elevated troponin and tachycardia suggest the possibility of **submassive PE** (PE associated with right ventricular dysfunction).
    - Elevated troponin levels may reflect myocardial stress, increasing the severity of submassive PE. Therefore, close monitoring and appropriate treatment are necessary.

2. **Anticoagulation Therapy:**
   - Anticoagulation is the first-line treatment for pulmonary embolism.
   - **Low Molecular Weight Heparin (LMWH):** Enoxaparin (1 mg/kg subcutaneously every 12 hours) is a commonly used option. Since the patient's kidney function is mildly reduced (eGFR 62 mL/min), no dose adjustment is typically required.
   - Alternatively, **direct oral anticoagulants (DOACs)** such as apixaban or rivaroxaban may be used. However, LMWH is preferred for initial treatment due to its rapid action.
3. **Oxygen Support:**
   - Oxygen therapy should be continued via nasal cannula, as the patient's oxygen saturation is 89%. The goal is to raise oxygen saturation above 90%.
4. **Thrombolytic Therapy (If Necessary):**
   - Although the patient is hemodynamically stable, the elevated troponin and potential right ventricular dysfunction warrant consideration of thrombolytic therapy. This is typically associated with submassive PE and should be carefully evaluated.
   - Thrombolytic therapy should be considered if the patient develops shock or significant hemodynamic instability.
5. **Evaluation of Right Ventricular Function:**
   - An echocardiogram should be performed to assess right ventricular function and detect any signs of right heart failure. If right ventricular dysfunction is present, the treatment plan may need to be adjusted accordingly.
6. **Patient Monitoring:**
   - Close monitoring is essential, particularly to watch for signs of right ventricular dysfunction. The first 24-48 hours are critical.
7. **Long-Term Treatment and Planning:**
   - Once stabilized, long-term anticoagulation should be planned for this patient. Anticoagulation therapy should continue for at least 3-6 months.
   - Management of comorbid conditions such as diabetes and hypertension is also important, as these may impact the course of treatment.

Conclusion: Initial treatment for this patient should include anticoagulation with low molecular weight heparin, oxygen support, and an echocardiographic evaluation of right ventricular function. Thrombolytic therapy should only be considered if hemodynamic instability develops.

Q15) A 36-year-old woman who is 32 weeks pregnant has been admitted to the hospital with an anterior STEMI. What is the most appropriate initial treatment for this patient?

A15) In this case, a 36-year-old woman who is 32 weeks pregnant has been admitted to the hospital with an anterior ST-elevation myocardial infarction (STEMI). The management of STEMI during pregnancy requires careful planning to ensure the safety of both the mother and the fetus. Anterior STEMI indicates significant myocardial damage, so rapid and effective treatment is essential.

Optimal Initial Treatment:

1. **Antiplatelet Therapy:**
   - **Aspirin:** As in standard STEMI management, aspirin (162-325 mg loading dose) should be administered. Aspirin is safe for both the mother and fetus and provides antiplatelet effects.

- **P2Y12 inhibitor (clopidogrel):** If PCI is planned, clopidogrel 300-600 mg loading dose should be given. Prasugrel and ticagrelor, stronger P2Y12 inhibitors, lack sufficient safety data during pregnancy, so clopidogrel is preferred.

2. **Anticoagulation:**
    - **Heparin:** Intravenous unfractionated heparin should be administered to provide anticoagulation during PCI. Low molecular weight heparin (LMWH) may also be considered, but intravenous unfractionated heparin is generally preferred during PCI.
3. **Reperfusion Therapy:**
    - **Primary PCI:** The gold standard for STEMI management during pregnancy is urgent primary percutaneous coronary intervention (PCI). It is the safest and most effective option for both the mother and fetus. The risk of fetal radiation exposure is minimal and can be further reduced with protective measures (e.g., lead shielding over the abdomen).
    - **Thrombolytic Therapy:** If PCI is not feasible, thrombolytic therapy (e.g., streptokinase or tenecteplase) is an alternative. However, thrombolytic therapy carries a higher risk of bleeding during pregnancy and should be considered only if PCI is not accessible.
4. **Nitrates:**
    - **Nitroglycerin** can be administered sublingually or intravenously, but care must be taken regarding hypotension or fetal risk. Nitrates are generally safe during pregnancy.
5. **Beta Blockers:**
    - Beta blockers are used to reduce myocardial oxygen demand and control ventricular rate. Cardioselective beta blockers such as metoprolol are preferred. However, care should be taken to avoid impairing uterine perfusion.
6. **Oxygen and Pain Management:**
    - If oxygen saturation is low, oxygen should be provided via nasal cannula.
    - **Morphine:** Can be used for pain control, but dosing should be cautious. The limited use of morphine in pregnancy is considered safe.
7. **Fetal Monitoring:**
    - While managing the mother's acute condition, the fetus should be continuously monitored. Fetal well-being should be assessed using cardiotocography (NST).

Conclusion: The optimal initial treatment for this patient includes antiplatelet therapy with aspirin and clopidogrel, anticoagulation with heparin, and primary PCI if possible. Nitrates and beta blockers can be used, but fetal monitoring should be maintained throughout. Thrombolytic therapy should only be considered if PCI is not available.

Q16) An 83-year-old male patient is admitted to the hospital with suspected acute aortic syndrome (AAS). The admitting team has requested several blood tests, including D-dimer. What is the role and use of D-dimer in the evaluation of patients with suspected acute aortic syndrome?

A16) Acute aortic syndrome (AAS), which includes aortic dissection, intramural hematoma, or aortic ulcer, is a life-threatening condition that requires prompt diagnosis and treatment. In this 83-year-old patient with suspected AAS, the D-dimer test and other imaging methods can play an important role in the diagnostic process.

Role of D-Dimer in the Evaluation of Acute Aortic Syndrome:

1. **Use of D-Dimer:**
    - D-dimer is a laboratory test that detects fibrin degradation products and is commonly used to rule out thrombotic events (pulmonary embolism, deep vein thrombosis). It can also aid in evaluating vascular events like aortic dissection.
    - In acute aortic syndrome, such as aortic dissection, D-dimer levels are elevated due to increased fibrin formation and breakdown. High D-dimer levels can support the suspicion of aortic dissection.
2. **Low D-Dimer Result:**
    - A negative or low D-dimer result can help rule out acute aortic syndrome, particularly in low to moderate-risk patients.
    - Literature suggests that when D-dimer levels are below 500 ng/mL, the likelihood of aortic dissection is significantly reduced. However, it is important to remember that a negative D-dimer does not entirely exclude aortic dissection. If clinical suspicion remains high, further imaging is required.
3. **High D-Dimer Result:**
    - Elevated D-dimer levels (>500 ng/mL) in patients, especially those presenting with acute chest or back pain, increase the suspicion of aortic dissection. However, D-dimer is a nonspecific test, meaning that levels can rise in various clinical conditions (e.g., infection, malignancy, pulmonary embolism).
    - If D-dimer is elevated, confirmatory imaging such as contrast-enhanced CT angiography, MRA, or transesophageal echocardiography should be performed to establish a definitive diagnosis.
4. **Limitations of D-Dimer:**
    - D-dimer levels tend to be elevated in older patients, which can lead to false-positive results due to age-related changes. Additionally, renal dysfunction, sepsis, or other factors may raise D-dimer levels, so it is not a standalone diagnostic tool and must be interpreted alongside clinical findings.

Conclusion: The D-dimer test is a rapid and non-invasive screening tool for patients with suspected acute aortic syndrome. If the D-dimer is negative and clinical suspicion is low, AAS can be ruled out. However, if D-dimer is elevated, urgent imaging (such as contrast-enhanced CT angiography or MRA) is essential to confirm or exclude aortic dissection or other serious conditions. Therefore, in cases of a positive D-dimer result, advanced imaging is mandatory for a definitive diagnosis.

Q17) A 69-year-old female patient presents to the hospital with sudden and severe central chest pain radiating to her back. The patient has had occasional chest pain in the past, but a coronary angiography performed three years ago revealed normal coronary arteries. During the examination, the patient reports mild epigastric pain. Her physical examination shows a heart rate of 84 bpm, blood pressure of 130/85 mmHg, and a mild systolic and diastolic murmur. There is no significant difference in blood pressure between the arms. The initial electrocardiogram (ECG) shows no ischemic changes and reveals normal sinus rhythm. Laboratory tests show a troponin level of 14 ng/L (normal <5 ng/L) and a D-dimer level of 1000 ng/mL (normal <500 ng/mL). Chest X-ray reveals mediastinal widening. What would be the most appropriate management strategy for this patient?

A17) In this case, a 69-year-old female patient presents with sudden and severe central chest pain radiating to the back. She has a history of occasional chest pain, but a coronary angiogram three years ago showed normal coronary arteries. The presence of a systolic and

diastolic murmur on physical examination, elevated D-dimer levels, and mediastinal widening on chest X-ray strongly suggest acute aortic dissection.

Optimal Management Strategy:

1. **Diagnosis of Acute Aortic Dissection:**
   - The nature of the chest pain, elevated D-dimer (1000 ng/mL), slightly elevated troponin (14 ng/L), and mediastinal widening are strong indicators of acute aortic dissection.
   - A normal ECG and mildly elevated troponin favor the diagnosis of aortic dissection over acute coronary syndrome.
2. **Urgent Imaging:**
   - **CT Angiography:** The first-choice imaging modality for confirming acute aortic dissection is contrast-enhanced thoracic CT angiography. This imaging will assess the location, extent, and urgency of the dissection.
   - If CT angiography is contraindicated, **transesophageal echocardiography (TEE)** is an alternative imaging option.
3. **Hemodynamic Management:**
   - Tight blood pressure control is crucial in aortic dissection management. The goal is to maintain a systolic blood pressure between 100-120 mmHg.
   - **Beta blockers:** First-line medications are beta blockers (e.g., intravenous esmolol or labetalol) because they reduce both heart rate and blood pressure, thereby decreasing stress on the aortic wall.
   - If blood pressure remains uncontrolled, **nitroprusside** or **calcium channel blockers** can be added, but beta blockers should be initiated first.
4. **Surgical Consultation:**
   - If the dissection involves the ascending aorta (**type A dissection**), this is a surgical emergency. Type A dissections are life-threatening and require urgent surgical repair.
   - If the dissection involves the descending aorta (**type B dissection**) and the patient is hemodynamically stable, conservative management (blood pressure control and close monitoring) is preferred. However, if complications arise (e.g., organ ischemia, rupture), surgical intervention may be necessary.
5. **Management of Possible Complications:**
   - Aortic dissection can lead to serious complications, including aortic rupture, tamponade, organ ischemia, and stroke. Prompt recognition and management of these complications are critical.

Conclusion: The optimal management strategy for this patient includes urgent confirmation of the dissection with CT angiography and strict hemodynamic control. If the dissection involves the ascending aorta, emergency surgical intervention is required. Beta blockers will be the mainstay of treatment for controlling blood pressure and heart rate during treatment.

Q18) A 39-year-old female patient with no known chronic medical conditions presents to the emergency department with complaints of palpitations and shortness of breath. Her blood pressure is measured at 92/54 mmHg. Physical examination reveals rales in the basal segments of both lungs and mild jugular venous pressure (JVP) elevation. An electrocardiogram (ECG) shows a wide QRS tachycardia. What would be the most appropriate management approach for this patient?

A18) In this case, a 39-year-old female presents to the emergency department with complaints of palpitations and shortness of breath. Her blood pressure is low (92/54 mmHg), an ECG

shows a wide QRS tachycardia, and physical examination reveals basal lung crackles and elevated jugular venous pressure. These findings suggest the patient is experiencing wide QRS tachycardia with hemodynamic instability, likely indicating ventricular tachycardia (VT) or another serious tachyarrhythmia.

Optimal Management Approach:

1. **Hemodynamic Stabilization:**
    - The patient has low blood pressure and appears hemodynamically unstable. Therefore, urgent electrical cardioversion should be considered.
    - **Synchronized Cardioversion:** If hemodynamic instability is present (e.g., hypotension, altered mental status, or severe shortness of breath), synchronized cardioversion should be performed promptly to treat the wide QRS tachycardia effectively. Cardioversion should be initiated at an energy level of 100-200 J.
2. **Antiarrhythmic Therapy (If Stabilized):**
    - If rhythm control is not achieved after cardioversion or the patient stabilizes, intravenous **amiodarone** can be initiated. Amiodarone is effective for treating wide QRS tachycardias and for controlling heart rate.
    - If amiodarone is not available or unsuitable, **lidocaine** may be considered as an alternative antiarrhythmic agent.
3. **Diagnostic Evaluation:**
    - The underlying cause of the wide QRS tachycardia should be investigated. While ventricular tachycardia is the most common cause, other conditions such as pre-excitation syndromes (e.g., Wolff-Parkinson-White syndrome) should be considered.
    - **Echocardiography** should be performed to evaluate cardiac function and structural abnormalities.
    - Electrolyte levels (especially potassium and magnesium) and cardiac enzymes should be checked.
4. **Fluid and Supportive Therapy:**
    - Intravenous fluids may be needed to improve the patient's low blood pressure. However, caution should be exercised as the patient may have signs of heart failure.
    - **Oxygen and High-Flow Support:** The findings of shortness of breath and jugular venous distention suggest possible pulmonary edema. Oxygen therapy and noninvasive mechanical ventilation (NIMV) should be provided if necessary.
5. **Monitoring and Follow-Up:**
    - The patient's cardiac rhythm and blood pressure should be continuously monitored, and preparations should be made for the recurrence of arrhythmias. An urgent consultation with a cardiologist is essential for further evaluation and management.

Conclusion: This patient is experiencing wide QRS tachycardia with hemodynamic instability, necessitating urgent synchronized cardioversion. Following cardioversion, antiarrhythmic therapy with amiodaron should be started, and the underlying cause of the tachycardia must be investigated.

Q19) A 78-year-old woman presents to the emergency department with chest pain, and an electrocardiogram (ECG) taken 10 minutes after her arrival shows signs of an inferior ST-elevation myocardial infarction (STEMI). The nearest primary percutaneous coronary

intervention (PCI) center is 130 minutes away by road. What would be the most appropriate management strategy for this patient?

A19) In this case, a 78-year-old woman presents to the emergency department with an inferior ST-elevation myocardial infarction (STEMI), and the nearest primary percutaneous coronary intervention (PCI) center is 130 minutes away. The goal in STEMI management is the rapid restoration of blood flow in the coronary arteries, making time a critical factor.

Optimal Management Strategy:

1. **Fibrinolytic Therapy:**
   - If access to primary PCI is not possible within 120 minutes, fibrinolytic therapy becomes the most appropriate option. Since the PCI center is 130 minutes away, fibrinolytic therapy should be initiated promptly rather than waiting.
   - Agents such as **alteplase**, **tenecteplase**, or **streptokinase** can be used. Fibrinolytic therapy should be administered as quickly as possible, ideally within the first 30 minutes, as time to treatment is directly related to patient outcomes.
2. **Antiplatelet and Anticoagulant Therapy:**
   - **Aspirin:** A loading dose of 160-325 mg should be given to chew.
   - **P2Y12 Inhibitor (clopidogrel):** A 300 mg loading dose should be administered. In the context of fibrinolytic therapy, clopidogrel is preferred over ticagrelor or prasugrel.
   - **Heparin or Low Molecular Weight Heparin (LMWH):** Anticoagulation should be initiated before or during fibrinolytic therapy. Unfractionated heparin (60 U/kg) or LMWH (e.g., enoxaparin) can be used.
3. **Transfer Planning:**
   - Once the patient has stabilized following fibrinolytic therapy, transfer to a primary PCI center should be arranged. If **rescue PCI** is required (e.g., in cases where fibrinolytic therapy is ineffective), the patient should be urgently transferred to the PCI center.
4. **Oxygen and Supportive Care:**
   - **Oxygen:** Should be administered if oxygen saturation is below 90%.
   - **Nitroglycerin:** Can be given sublingually, provided there is no hypotension (blood pressure should be >90 mmHg).
   - **Morphine:** May be used for pain relief and to reduce anxiety but should be monitored closely as it can cause hypotension.

Conclusion: The optimal management for this patient involves rapid initiation of fibrinolytic therapy, as access to PCI exceeds the 120-minute threshold. After fibrinolytic therapy, the patient should be transferred to a PCI-capable center. Antiplatelet therapy with aspirin and clopidogrel, along with anticoagulation using heparin or LMWH, should be administered.

Q20) A 53-year-old male patient presents to the emergency department with acute inferior myocardial infarction (MI). His heart rate is 85 beats per minute, and his blood pressure is 92/58 mmHg. No murmurs or crackles are heard on auscultation, but his jugular venous pressure (JVP) is measured at 14 cmH2O. What would be the most appropriate treatment to manage the low blood pressure in this patient?

A20) In this case, a 53-year-old male presents with an acute inferior myocardial infarction (MI) and low blood pressure (92/58 mmHg). Inferior MI is often associated with right

ventricular infarction, which can lead to low blood pressure. The elevated jugular venous pressure (14 cmH2O) further suggests the possibility of right heart failure. The patient's normal heart rate (85 bpm) supports this diagnosis as well.

Management of Low Blood Pressure:

1. **Rule Out Right Ventricular Infarction:**
   - In patients with inferior MI, right ventricular infarction is common. Patients with right ventricular infarction often require aggressive fluid resuscitation, as the goal is to increase ventricular filling pressures due to impaired right ventricular preload.
   - Echocardiography should be performed to assess right ventricular function.
2. **Intravenous Fluid Resuscitation:**
   - If hypotension is due to right ventricular infarction or low preload, volume resuscitation with isotonic saline should be initiated. This can help increase right ventricular filling and stabilize blood pressure.
   - Start with 250-500 mL of isotonic saline rapidly, and continue based on the patient's response. The patient's response to fluids should be monitored closely.
3. **Inotropic Support (If Necessary):**
   - If fluid resuscitation alone does not improve blood pressure, inotropic agents may be required. **Dobutamine** can be used to improve right ventricular contractility and enhance blood pressure.
   - Inotropic support is particularly important in cases of impaired right ventricular function.
4. **Avoid Vasodilators:**
   - Vasodilators such as nitroglycerin should be avoided in patients with right ventricular infarction. These drugs can reduce preload, further decreasing right ventricular filling and exacerbating hypotension.
5. **Other Treatment Considerations:**
   - The patient should continue receiving antiplatelet therapy with **aspirin** and a **P2Y12 inhibitor**. If PCI is planned, anticoagulation therapy (e.g., heparin) should also be administered.
   - Oxygen therapy should be provided if the oxygen saturation is below 90%.

Conclusion: The most appropriate approach for this patient involves initiating intravenous fluid resuscitation, taking into account the possibility of right ventricular infarction. If fluid therapy is insufficient, inotropic support should be considered. Vasodilators such as nitroglycerin should be avoided, and fluid resuscitation should be carefully monitored.
